# Lateral Internal Anal Sphincterotomy of Chronic Anal Fissure: An Experience of 165 Cases

**DOI:** 10.7759/cureus.30530

**Published:** 2022-10-20

**Authors:** Amer F AL-Ubaide, Sami M Al-Rubaye, Raid M Al-Ani

**Affiliations:** 1 Department of Surgery/General Surgery, University of Anbar, College of Medicine, Ramadi City, IRQ; 2 Department of Surgery, Al-Muqdadiya General Hospital, Diala Health Directorate, Diala City, IRQ; 3 Department of Surgery/Otolaryngology, University of Anbar, College of Medicine, Ramadi City, IRQ

**Keywords:** flatus incontinence, fecal incontinence, lateral internal anal sphincterotomy, anal fissure, chronic anal fissure

## Abstract

Background: The anal fissure is frequently seen in surgical practice. It is caused by a longitudinal tear in the anoderm distal to the dentate line. The hallmark feature of the disease is severe pain during defecation. Chronic anal fissure (CAF) causes undue stress that leads to poor quality of life. Different options of treatment, whether medical or surgical, are employed to treat this condition. One of these modalities is lateral internal anal sphincterotomy (LIAS).

Objectives: We aimed to assess the safety and efficacy of the LIAS surgical procedure for the treatment of CAF.

Materials and methods: This retrospective study was conducted at the Al-Muqdadiya General Hospital, Diala, Iraq, for a period from January 2016 to March 2021. The medical records of the patients with CAF who were subjected to LIAS under local anesthesia were reviewed. Data regarding the age, gender, smoking habit, body mass index (BMI), healing time, complications, and outcome for each participant were recorded.

Results: Of 165 participants, there were 91 men. The majority of the cases were ≤ 35 years (78.19%), were non-smokers (80%), and had no history of DM (98.79%). There was complete healing with a resolution of the pain at two months postoperatively in 163 subjects. The most common complication in our study was flatus incontinence (n = 5). All of them were found in the age group > 35 years and women. There were statistically significant differences between the age groups, gender, BMI, and the occurrence of flatus incontinence (P-value = 0.000, 0.012, and 0.009 respectively). However, there was no such association between smoking habit and a history of DM (P-value > 0.05).

Conclusion: LIAS is safe and effective in the treatment of CAF, with an excellent outcome in resolving pain and a low complication rate. Age, female gender, and high BMI might affect the occurrence of flatus incontinence.

## Introduction

An anal fissure is a common problem seen in daily surgical practice [1]. It can be defined as a tear in the anoderm distal to the dentate line. The pathophysiology of anal fissures is thought to be related to trauma from either the passage of hard stool or prolonged diarrhea. A tear in the anoderm causes spasms of the internal anal sphincter, which results in pain, increased tearing, and decreased blood supply to the anoderm. The sequence of pain, spasm, and ischemia contribute to the development of a poorly healing wound that becomes a chronic anal fissure (CAF), usually located in the posterior midline, with visible sphincter fibers at the base of the fissure, anal papillae, sentinel piles, and indurated margins [2]. The tearing pain with defecation is the chief complaint followed by rectal bleeding, tenesmus, constipation, and pruritus [3].

The treatment options focus on breaking the chain of pain, spasm, and ischemia, which is thought to be responsible for the development of fissures in ano. In acute fissures, healing can be achieved by a variety of conservative treatments [4]. But the same treatment is not successful in treating a CAF. A variety of surgical treatment option techniques have been developed for the management of CAFs. Lateral internal anal sphincterotomy (LIAS) is one of the most practiced treatments for CAFs and can be effective in more than 90% of cases. Nonetheless, it has its complications; the chief complication after this surgery is incontinence to feces or flatus, which has been reported in the range of 3.3-7% [5]. LIAS can be performed with either local anesthesia or sedation [6].

We aimed to assess the effectiveness and safety of the LIAS for the treatment of CAF.

## Materials and methods

A retrospective study was conducted at Al-Muqdadiya General Hospital, Dialya City, Iraq, in the period between January 2016 to March 2021. One hundred sixty-five patients with CAF for six months or more, who were not responsive to conservative therapy or subjected to the previous surgical operation were included in this study; all of them underwent LIAS. A chronic fissure was distinct as a presented lesion with indurated edges, sentinel piles, with or without hypertrophied anal papillae, and the existence of circular muscle fibers at the base of the fissure. Pregnant women, patients with associated diseases like hemorrhoids, abscesses, and fistula, those with previous anal surgery, and those who escaped follow-up were excluded from the current study.

Full history and complete examination were done in addition to a local exam regarding the sites of the fissure, indurations, and skin tags. Data regarding age, gender, smoking habit, body mass index (BMI), response to treatment concerning pain resolution at one week and two months, follow-up concerning the healing time, complications, and outcome for each participant were recorded. Preoperative rectal enemas or prophylactic antibiotics were not used. All operations were performed with a strict aseptic technique under local anesthesia with lidocaine (40-80 mg).

Data were entered and analyzed using IBM SPSS Statistics for Windows, Version 26.0 (Released 2019; IBM Corp., Armonk, New York, United States). The continuous variables were presented as mean ± SD. While the categorical variables were expressed as frequencies and percentages. A Chi-square test was used to compare the categorical variants. An independent t-test was used to compare the means. A p-value of less than 0.05 was considered a statistically significant difference. The present study was approved by the Ethical Approval Committee of the University Of Anbar, Ramadi City, Anbar, Iraq (approval no 145).

## Results

**Figure 1:**
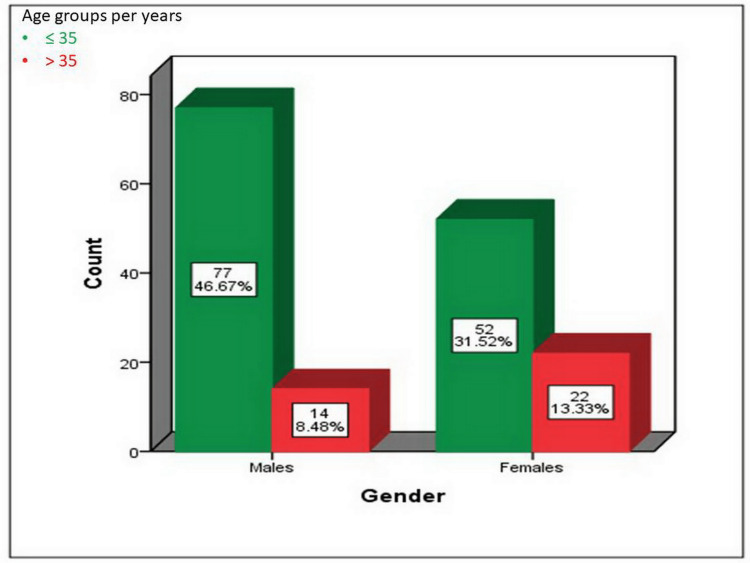
The distribution of the 165 patients regarding age and gender. P-value = 0.026.

**Figure 2:**
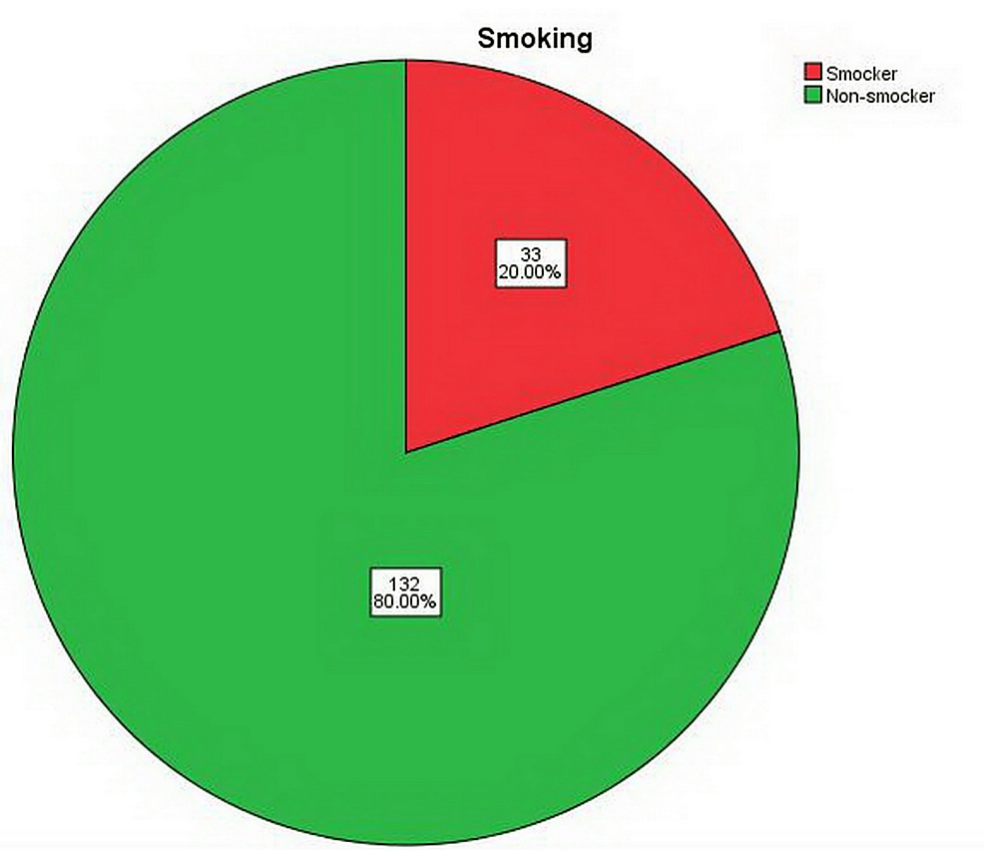
The distribution of the 165 patients according to their smoking habits.

**Figure 3:**
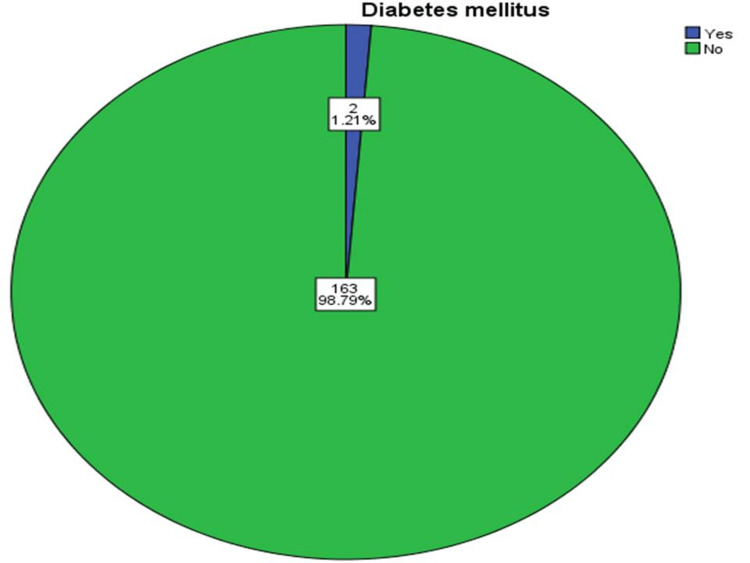
The distribution of the 165 patients according to the history of diabetes mellitus.

Out of 165 participants, there were 91 males. The majority of the cases belong to the age group ≤ 35 years (46.67% male and 31.52% female). There was a statistically significant difference between the age group and gender regarding the occurrence of CAF (p-value = 0.026) as shown in Figure 1. The mean age of our patients was 29.16 ± 3.67 years (age range 20-46 years). The majority of the subjects were non-smokers (n = 132, 80%) and had no history of DM (n = 163, 98.79%) (Figures 2, 3).

Table 1 shows the excellent outcomes for the patients concerning pain resolution at one week and two months and healing at two months.

**Table 1:** The outcome of the 165 patients following lateral internal anal sphincterotomy.

Outcomes	Frequency	Percentages
Pain resolution at one week	165	100
Pain resolution at two months	163	98.8
Healing at two months	163	98.8

The most common complication in our study was flatus incontinence (n = 5, 3%) while there was no fecal incontinence in any case (Table 2).

**Table 2:** The complications in the 165 patients following lateral internal anal sphincterotomy.

Complications	Frequency	Percentages
Flatus incontinence	5	3
Bleeding	4	2.4
Infection	2	1.2
Fecal incontinence	0	0

All five cases with flatus incontinence were found in the age group > 35 years and were females and non-smokers. There were statistically significant differences between age group and gender in the occurrence of flatus incontinence (p-value = 0.0001 and 0.012, respectively). However, there was no such association with the smoking habit (p-value = 0.256). The mean BMI of the participants was 29.38 ± 2.76 kg/m^2^ (range 26.01-39.65). There was a statistically significant difference between the BMI and the occurrence of flatus incontinence (p-value = 0.009) while there was no such association between the patients with and without DM and the occurrence of flatus incontinence (Table 3). 

**Table 3:** The relationship between the occurrence of flatus incontinence and certain variables in the 165 patients.

Variables	Flatus incontinence			P-value
	Yes 5 (3%)	No 160 (97%)	Total 165 (100%)	
Age groups per years				0.0001
≤ 35	0 (0)	129 (100)	129 (100)	
> 35	5 (13.9)	31(86.1)	36 (100)	
Gender				0.012
Males	0 (0)	91 (100)	91 (100)	
Females	5 (6.8)	69 (97)	74 (100)	
Smoking				0.256
Smoker	0 (0)	33 (100)	33 (100)	
Non-smoker	5 (3.8)	127 (96.2)	132 (100)	
Mean BMI (kg/m^2^)	32.53 ± 4.24	29.27 ± 2.66		0.009
Diabetes mellitus				0.801
Yes	0 (0)	2 (100)	2 (100)	
No	5 (3.1)	158 (96.9)	163 (100)	

## Discussion

Anal fissures are caused by spasms of the anal muscles and can cause anal pain that can be quite severe, usually during and after a bowel movement [7]. There are no clear guiding principles on anal fissure management. The management purpose is to break the cycle of anal sphincter spasm to improve the blood perfusion to the area, which will lead to the healing of the fissure [8]. There are various management choices for CAFs, but the conformity of which is the best modality of treatment is not yet settled. Conservative options may be used without risking incontinence. However, recurrence is common in these groups of patients in addition to the incidence of medication side effects [9]. The surgical option is an efficient method of reducing internal anal sphincter spasms. Nonetheless, it has its complications. The chief complication after this surgery is incontinence to feces or flatus, which has been reported in the range of 3.3-7% [5]. LIAS is often claimed to be the gold standard therapy for CAFs for its high efficiency compared with other therapies, particularly medical treatment [10]. LIAS can be performed with either local anesthesia or sedation [6]. In our study, we found that LIAS is safe and effective in treating patients who have a CAF.

In the current study, the mean age was 29.16 years while male to female ratio was 1.2 :1 with a slight predominance of men; this is because most female patients attend gynecology clinics due to religious and cultural habits and it is compatible with a study done by Latif et al. in 2013, which had a mean age of 30 years and male to female ratio of 1.8:1 [11].

It is well known that bleeding, wound infection, and abscesses are considered general complications following every operation [12]. Incontinence to gas and stool has emerged as a major concern after sphincterotomy [1]. Incontinence rates of up to 14% have been reported, but these vary widely among studies [13]. Much of this variation can be attributed to differences in definition and assiduousness of follow-up. Incontinence is the main complication feared by most surgeons [1]. We think that an accurate operation with a correct indication of surgery should avoid this problem. Although complications such as infection, abscess, fistula, and hematoma can be seen in the early period after LIAS, they occur only in rare cases [14], as seen in our study (four cases with bleeding and two cases with infection). Fecal incontinence was not observed in the current study. However, the study revealed that 3% of patients above 35 years had developed flatus incontinence. All of them were females, multiparous, delivered vaginally, and they had impending incontinence due to the mechanical damage of the anal sphincter in first labor and subsequent delivery that was the trigger of incontinence or increased its severity, especially flatus post LIAS, which may correlate the increased incidence of anal incontinence in vaginally delivered mothers. This observation was noted in previous studies [15-17]. Furthermore, in the present study, three of those females had a history of episiotomy procedures and this may be a risk factor for anal incontinence. Two previous investigations demonstrated that studies have highlighted an association between adolescent pregnancies and increased incidence of risk factors for fecal incontinence, such as assisted deliveries, the prolonged second stage of labor, and perineal tears [18,19]. In a study done by Ahmad and Aziz in 2016, they found that 3.3% of their patients who underwent close LIAS have flatus incontinence [20], which is compatible with our study. Also, 2% of patients had temporary incontinence in a study done by Akhtar et al. from Pakistan in 2018 [21].

Smoking and diabetes are risk factors for fecal incontinence as was reported in Townsend et al.ʼs study in 2013 [22]. They found that women who currently smoked 25 cigarettes per day had 1.5-fold higher odds of fecal incontinence compared with never smokers. Type 2 diabetes, high blood pressure, and neurologic disease were associated with 1.2-1.7-fold increased odds of fecal incontinence. However, the present study didn't find a significant correlation between smoking habit and diabetes with flatus incontinence, which may be due to sampling paucity for these variables.

A significant relationship was found between obesity (BMI 32.53 kg/m^2^ or more) and flatus incontinence (p-value = 0.009), and it was found that higher BMI was significantly associated with leakage of liquids in women with BMI ≥ 35 kg/m^2^ [22]. In another research conducted by Erekson et al. in 2008, they found that increasing BMI is significantly associated with anal incontinence, but not defecatory dysfunction in women [23].

The small sample size was considered a limitation of the present study and its results cannot be generalizable in clinical practice. Another limitation was the retrospective nature of the study. Thirdly, there was no provision of anal manometer in our hospital to monitor anal pressure.

## Conclusions

The LIAS procedure is very effective and safe in the treatment of CAF owing to high efficacy and low complications. Multiparous obese patients had a relatively high rate of flatus incontinence. Flatus incontinence did not persist for a long time.
